# A Novel Function of Human Pumilio Proteins in Cytoplasmic Sensing of Viral Infection

**DOI:** 10.1371/journal.ppat.1004417

**Published:** 2014-10-23

**Authors:** Ryo Narita, Kiyohiro Takahasi, Etsu Murakami, Emi Hirano, Seiji P. Yamamoto, Mitsutoshi Yoneyama, Hiroki Kato, Takashi Fujita

**Affiliations:** 1 Laboratory of Molecular Genetics, Institute for Virus Research, Kyoto University, Kyoto, Japan; 2 Institute for Innovative NanoBio Drug Discovery and Development, Graduate School of Pharmaceutical Science, Kyoto University, Kyoto, Japan; 3 Division of Molecular Immunology, Medical Mycology Research Center, Chiba University, Chiba, Japan; 4 Laboratory of Molecular Cell Biology, Graduate School of Biostudies, Kyoto University, Kyoto, Japan; Harvard Medical School, United States of America

## Abstract

RIG-I-like receptor (RLR) plays a pivotal role in the detection of invading pathogens to initiate type I interferon (IFN) gene transcription. Since aberrant IFN production is harmful, RLR signaling is strictly regulated. However, the regulatory mechanisms are not fully understood. By expression cloning, we identified Pumilio proteins, PUM1 and PUM2, as candidate positive regulators of RIG-I signaling. Overexpression of Pumilio proteins and their knockdown augmented and diminished IFN-β promoter activity induced by Newcastle disease virus (NDV), respectively. Both proteins showed a specific association with LGP2, but not with RIG-I or MDA5. Furthermore, all of these components were recruited to NDV-induced antiviral stress granules. Interestingly, biochemical analyses revealed that Pumilio increased double-stranded (ds) RNA binding affinity of LGP2; however, Pumilio was absent in the dsRNA-LGP2 complex, suggesting that Pumilio facilitates viral RNA recognition by LGP2 through its chaperon-like function. Collectively, our results demonstrate an unknown function of Pumilio in viral recognition by LGP2.

## Author Summary

Mammals utilize innate immune system to counteract viral infections. The host pattern-recognition receptors, such as RIG-I-like receptors (RLRs), sense invading pathogens and initiate innate immune responses. RLRs are composed of three RNA helicases, RIG-I, MDA5 and LGP2, and detect a series of RNA viruses, such as influenza or hepatitis C virus, in the cytoplasm. Upon RNA virus infection, RLRs transmit signals through mitochondrial adaptor protein, IPS-1, to activate transcription factor IRF-3/7, resulting in the production of type I interferon (IFN). Type I IFN plays a crucial role in innate immune system by inducing a hundreds of interferon-stimulated genes and its induction is tightly controlled at transcriptional and translational steps. Pumilio proteins are originally identified as translational repressor through direct binding to specific sequence motifs in the 3′ untranslated regions of specific mRNA, and regulate critical biological processes, such as development and differentiation. In this report, we identified human Pumilio proteins, PUM1 and PUM2, as candidate regulators of IFN signaling. Our results demonstrated an unknown function of Pumilio in viral recognition by LGP2.

## Introduction

The host innate immune system is the first line of defense against invading pathogens. Pattern-recognition receptors (PRRs) detect pathogen molecules, termed pathogen-associated molecular patterns (PAMPs), to initiate innate immune responses [1], [2], [3], [4], [5]. Viruses invade host cells to replicate their genome and produce new infectious virions. RIG-I-like receptors (RLRs), including RIG-I, MDA5 and LGP2, sense the invasion and generation of viral RNA PAMPs and trigger antiviral responses [6], [7]. In the resting state, RIG-I and MDA5 exist in an autorepressed state, in which N-terminal caspase activation and recruitment domains (CARDs) are masked by the helicase domain; however, upon virus infection, these helicases are activated and oligomerized along with RNAs to form filament-like structures [8], [9]. Signals from RLRs are relayed to an adaptor, IPS-1 (also known as MAVS, VISA, Cardif) [10], [11], [12], [13], [14], [15], which then recruits TRAF adaptors, protein kinases TBK-1, IKK-i and IKK complex to activate transcription factors IRF-3, -7 and NF-κB [16], [17].

Knockout mouse studies have shown that RIG-I and MDA5 play a pivotal role in the detection of a series of RNA viruses in vivo [18]. RIG-I detects Sendai virus, NDV and influenza A virus, whereas viruses belong to picornaviridae are sensed by MDA5. Although the mechanism underlying the differential sensing of different viruses by RIG-I and MDA5 is not completely understood, it is proposed that virus specificity comes from the dsRNA length and 5′-end structure of viral RNA [19], [20], [21], [22]. LGP2 was originally thought to be a negative regulator because it lacks CARD, which is crucial for signal transduction. However, knockout and knock-in mouse studies have shown that LGP2 functions as a positive regulator via its ATPase activity [23], consistent with its high affinity binding with dsRNA [7], [24].

Recent studies have reported that RLR signaling is subject to numerous regulations [25]. TRIM25 positively regulates signaling through interactions with RIG-I and ubiquitination [26]. Riplet (also termed RNF135 and REUL) positively regulates RIG-I signaling through ubiquitination of RIG-I, independent of TRIM25 [27], [28]. On the other hand, ubiquitin ligases, RNF125 [29] and A20 [30], and deubiquitinating enzymes, DUBA [31] and CYLD [32], are reported to function as negative regulators of RIG-I signaling. In addition to the ubiquitination of signaling peptides, involvement of the free ubiquitin chain has been proposed [33]. Furthermore, accumulating reports suggest the importance of the virus-induced stress response in antiviral innate immunity. In particular, viral infection induces antiviral stress granules (avSGs), including RIG-I, MDA5, LGP2 and viral RNA [34], [35], [36], [37].

Our expression cloning for antiviral signal regulators identified Pumilio proteins. Pumilio proteins (also termed PUF, Pumilio/FBF) are evolutionary conserved from plants to mammals and were originally identified as translational repressors through direct binding to the specific sequence termed the Nanos response element (NRE) present within the 3′-UTR of target mRNAs, thereby regulating various processes: embryonic development, stem cell differentiation, cell cycle and mitochondrial biogenesis [38], [39], [40], [41], [42]. In this report, we describe a novel and non-translational function of Pumilio proteins in viral recognition by LGP2.

## Results

### Pumilio Proteins Positively Regulate RIG-I Signaling in Response to NDV Infection

**Figure 1:**
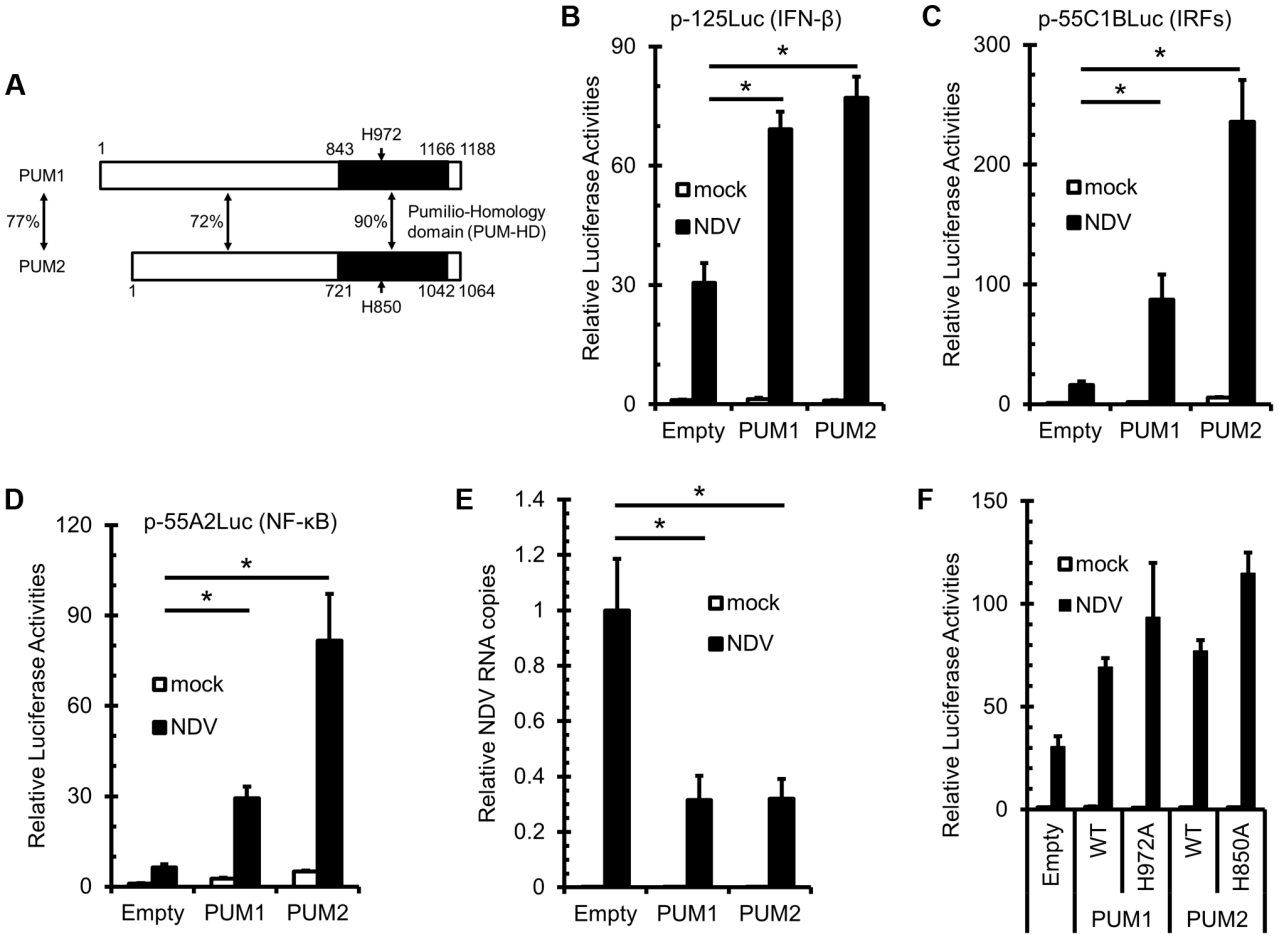
Overexpression of PUM1 and PUM2 results in enhanced NDV-induced *IFNB* promoter activity. (A) Schematic representation of PUM1 and PUM2. PUM-HD shows high sequence similarity between PUM1 and PUM2. Positions of histidine (H) residues critical for NRE recognition are indicated. (B–D) L929 cells were transfected with the indicated reporter gene, p-125Luc (B), p-55C1BLuc (C) or p-55A2Luc (D), and pRL-tk, together with the expression vector for PUM1 or PUM2. The cells were stimulated by NDV infection for 9 h and subjected to a dual-luciferase assay. (E) L929 cells were transfected with an expression vector for PUM1 or PUM2. The cells were infected with NDV for 24 h, and then NDV RNA levels were determined by quantitative RT-PCR. (F) L929 cells were transfected with p-125Luc and pRL-tk, together with the expression vector for wt and histidine mutants of PUM1 or PUM2 as indicated. The cells were stimulated by NDV infection and subjected to a dual-luciferase assay. Data are from one representative of at least two independent experiments; means and S.D. of duplicate experiments are shown (*p<0.05).

We previously identified RIG-I by expression cloning of the human cDNA library using virus-inducible reporter gene activity as the readout [6]. This strategy allowed us to identify other candidate regulators of antiviral signaling, Pumilio proteins. Pumilio proteins share a highly conserved C-terminal Pumilio-homology domain (PUM-HD) [43]. In humans, two genes encoding PUM1 and PUM2 exist. Human PUM1 and PUM2 have similar domain structures and have high homology in their primary structure (Fig. 1A). It was reported that PUM-HD is responsible for sequence-specific RNA binding, whereas the function of the N-terminal portion is unknown. We obtained two independent clones encoding full-length PUM1 and one clone of PUM2 (missing coding amino acids 1-368) by expression cloning. We constructed expression vectors for full-length PUM1 and PUM2 and examined their effect on *IFNB* promoter activity in L929 cells. Overexpression of PUM1 and PUM2 augmented *IFNB* promoter activity induced by NDV infection (Figure 1B). As *IFNB* promoter is regulated by both IRFs and NF-κB, we investigated whether PUM1 and PUM2 affect promoter activity regulated by IRFs (p-55C1B) and NF-κB (p-55A2). PUM1 and PUM2 augmented p-55C1BLuc activity (Figure 1C), as well as p-55A2Luc activity (Figure 1D), suggesting that PUM1 and PUM2 mediate the activation of both IRFs and NF-κB transcription factors. In accord with the increased IFN promoter activity, NDV RNA replication was suppressed by the overexpression of PUM1 and PUM2 24 h after NDV infection, suggesting that PUM1 and PUM2 share antiviral potential (Figure 1E). It is known that translational repression by PUM-HD depends on H850 in PUM2 [44] and this residue was conserved between PUM1 (H972) and PUM2. Therefore, we constructed expression vectors for their alanine mutants and tested their antiviral activity. Interestingly, these mutants markedly enhanced NDV-induced *IFNB* promoter activity (Figure 1F), suggesting that the enhancing function is independent of the translational repression function of Pumilio proteins.

**Figure 2:**
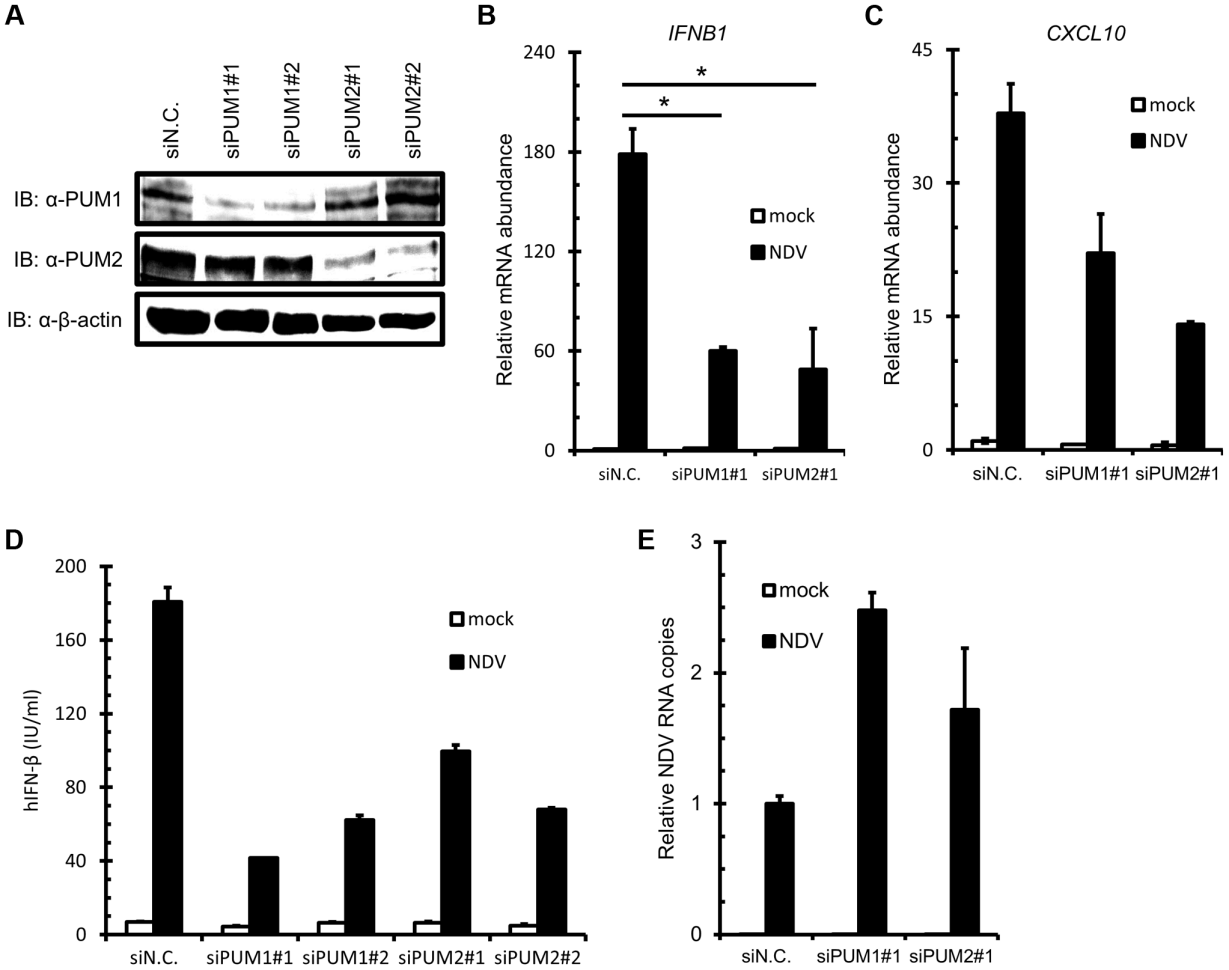
Knockdown of PUM1 and PUM2 downregulates NDV-induced gene activation. (A–C) HEK293T cells were transfected with control siRNA (siN.C.) or siRNA targeting human PUM1 or PUM2 for 48 h. Knockdown efficiency was confirmed by immunoblotting with anti-PUM1, anti-PUM2 and anti-β-actin antibodies (A). The cells were infected with NDV for 9 h, and *IFNB* (B) and *CXCL10* (C) mRNA levels were determined by quantitative RT-PCR. (D and E) HEK293T cells were transfected with control siRNA or siRNA targeting PUM1 or PUM2 for 48 h. The cells were infected with NDV for 24 h. The culture media were collected and subjected to IFN-β ELISA (D). Total cellular RNA was extracted and subjected to qRT-PCR for NDV RNA (E). Data are from one representative of at least two independent experiments; means and S.D. of duplicate experiments are shown (*p<0.05, **p<0.01).

To further investigate the function of PUM1 and PUM2 in IFN induction, we performed siRNA-mediated knockdown. siRNA targeting for human PUM1 and PUM2 suppressed the expression of endogenous PUM1 and PUM2 protein, respectively (Figure 2A). As expected, knockdown of endogenous PUM1 or PUM2 impaired the mRNA expression of *IFNB1* and *CXCL10*, one of the IFN-stimulated genes, in response to NDV infection (Figure. 2B and C). We also examined IRF-3 phosphorylation in the PUM knockdown cells, as well as IRF-3 dimerization. As shown in Figure S1A and B, both phosphorylation and dimerization of IRF-3 were impaired in the Pumilio knockdown cells. Furthermore, the production of IFN-β protein was also reduced by PUM1 or PUM2 knockdown upon NDV infection (Figure 2D). Conversely, NDV RNA copies were increased by knockdown of PUM1 or PUM2 (Figure 2E). To rule out the possibility that Pumilio proteins regulate NDV-induced IFN production through affecting the expression level of RLRs, we examined the basal level of RLRs in the Pumilio knockdown cells. The knockdown of Pumilio proteins did not affect the basal and IFN-induced expression level of RLRs (Figure S2). Finally, we also tested synthetic oligonucleotides, such as poly I:C, *in vitro*-transcribed 5′pppRNA and poly dA:dT. In contrast to NDV infection, the knockdown of Pumilio proteins did not affect the IFN production in response to these stimuli (Figure S1C). These results suggest that PUM1 and PUM2 positively regulate antiviral responses against NDV by controlling IFN production.

### Physical Association of PUM1 and PUM2 with LGP2

**Figure 3:**
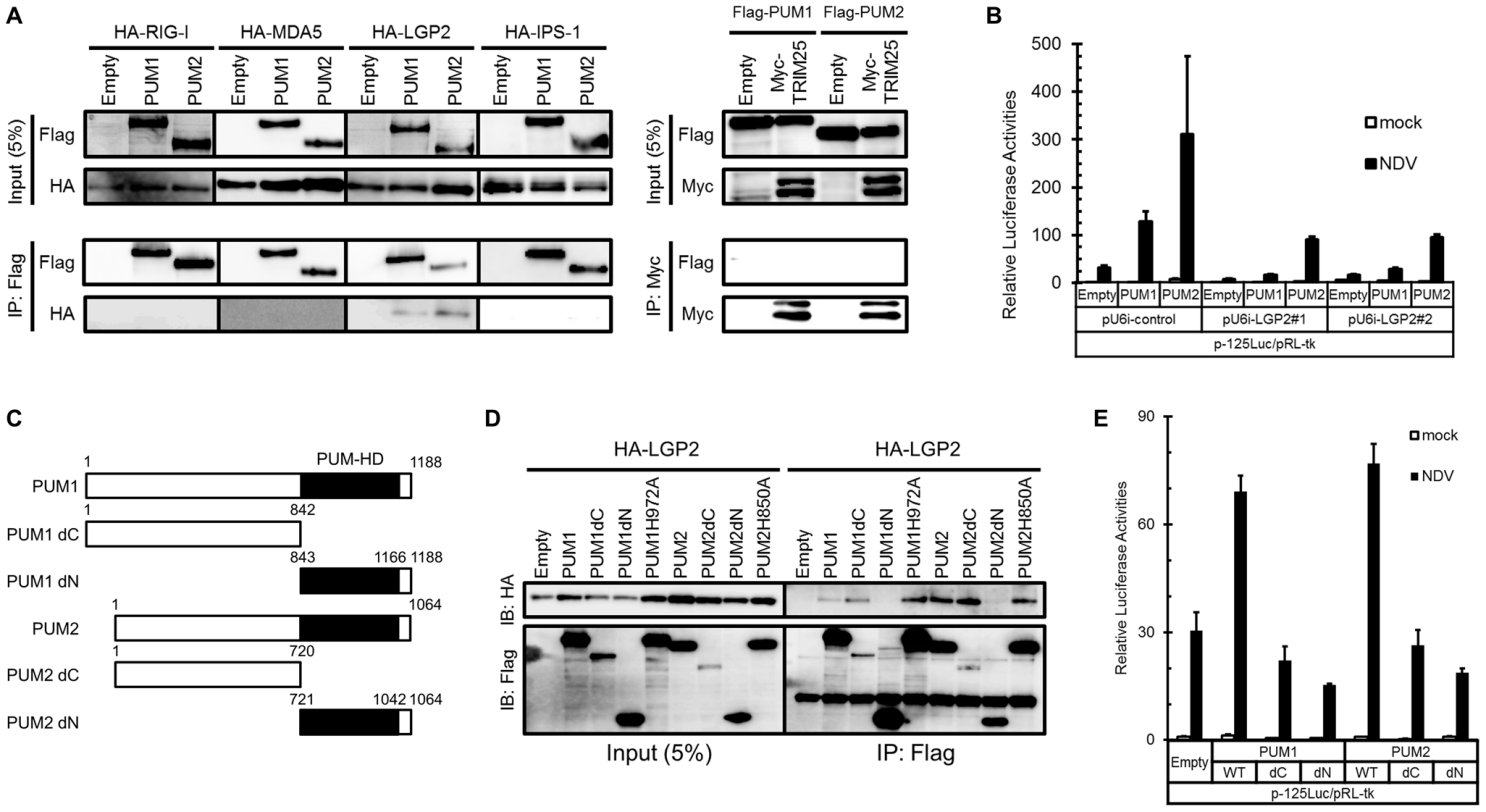
Physical association of PUM1 and PUM2 with LGP2 and involvement of N- and C-terminal domains of PUM1 and PUM2 in IFN induction. (A) HEK293T cells were transfected with expression vector HA-tagged RIG-I, MDA5, LGP2 or IPS-1, together with Flag-tagged PUM1 or PUM2. For TRIM25, HEK293T cells were transfected with Flag-tagged PUM1 or PUM2, together with Myc-tagged TRIM25. The cell lysates were subjected to anti-Flag or anti-c-Myc immunoprecipitation (IP), followed by Western blotting. Western blotting result of total lysate is shown as a reference (Input, 5%). (B) L929 cells were transfected with control shRNA construct (pU6i-control) or shRNA for LGP2 (pU6i-LGP2#1 and #2) and expression vectors for PUM1 or PUM2 and p-125Luc reporter and pRLtk as indicated. The cells were stimulated by infection with NDV for 9 h and subjected to the dual luciferase assay. (C) Schematic representation of PUM1 or PUM2 deletion mutants used for IP experiments. (D) HEK293T cells were transfected with expression vectors for the indicated proteins and for HA-tagged LGP2. The cell lysates were subjected to IP with anti-Flag, followed by immunoblotting with anti-HA. Immunoblotting result of total lysate is shown as a reference (Input, 5%). (E) L929 cells were transfected with expression vectors for the wild type or mutant of PUM1 or PUM2 and p-125Luc reporter and pRL-tk as indicated. Cells were stimulated by infection with NDV for 9 h and subjected to the dual luciferase assay. Data are from one representative of at least two independent experiments (means and s.d. of duplicate experiments.)

It has been shown that NDV infection is mainly detected by one of the RLRs, RIG-I [18]. TRIM25 regulates the activation of RIG-I through ubiquitination of RIG-I [26]. In addition, IPS-1 is an adaptor protein essential for RLR signaling [14], [15]. To elucidate the regulatory mechanism, the physical association of full-length PUM1 and PUM2 with RLRs, TRIM25 and IPS-1 was examined by co-immunoprecipitation. As shown in Figure 3A, LGP2, but not RIG-I nor MDA5 was precipitated with PUM1 or PUM2. No interaction of PUM1 and PUM2 with TRIM25 and IPS-1 was detectable, indicating that PUM1 and PUM2 selectively interact with LGP2. We also investigated whether PUM1 and PUM2 interacted with each other. As shown in Figure S3, PUM2 associated with PUM1. This result suggested that PUM1 and PUM2 exist as heteromeric complex.

LGP2 was shown to function as a positive regulator of RIG-I- and MDA5-mediated antiviral responses [23]. Specific associations between LGP2 and PUM1 and PUM2 prompted us to investigate the involvement of LGP2 in Pumilio-mediated transactivation. L929 cells were transfected with the expression vector for shRNA either non-targeted or targeted to LGP2, then transactivation by PUM1 or PUM2 was examined (Figure 3B). Knockdown of LGP2 markedly attenuated transactivation by PUM1 or PUM2, suggesting that the observed physical interaction between LGP2 and PUM1 and PUM2 is relevant to the biological activity of these regulators.

To elucidate the involvement of C-terminal PUM-HD in the association between LGP2 and PUM1 and PUM2, expression vectors for PUM-HD (PUM1dN and PUM2dN) and the rest (PUM1dC and PUM2dC) were constructed (Figure 3C). Co-immunoprecipitation using the mutants revealed that LGP2 interacted with PUM1 and PUM2 lacking PUM-HD as strongly as with the respective full-length proteins, while interaction with PUM-HD (dN constructs) was undetectable. Consistent with the lack of interaction between PUM-HD and LGP2, PUM1H972A and PUM2H850A efficiently co-precipitated with LGP2 (Figure 3D). We also determined the domain of LGP2 responsible for the interaction with Pumilio proteins. As shown in Figure S4, LGP2 helicase domain is important for LGP2 to interact with Pumilio proteins. These results suggest that PUM1 and PUM2 interact with LGP2 through the N-terminal domain.

### Involvement of Both N- and C-Terminal Domains of PUM1 and PUM2 in the Activation of Antiviral Response

To further elucidate the mechanism of transactivation by PUM1 and PUM2, full-length and dN and dC mutants were tested for *IFNB* promoter activation (Figure 3E). Full-length PUM1 and PUM2 but neither dC nor dN enhanced NDV-induced IFN-β reporter activity, indicating that both PUM-HD and the N-terminal portion are necessary for transactivation.

### Co-localization of PUM1 and PUM2 in avSGs upon NDV Infection

**Figure 4:**
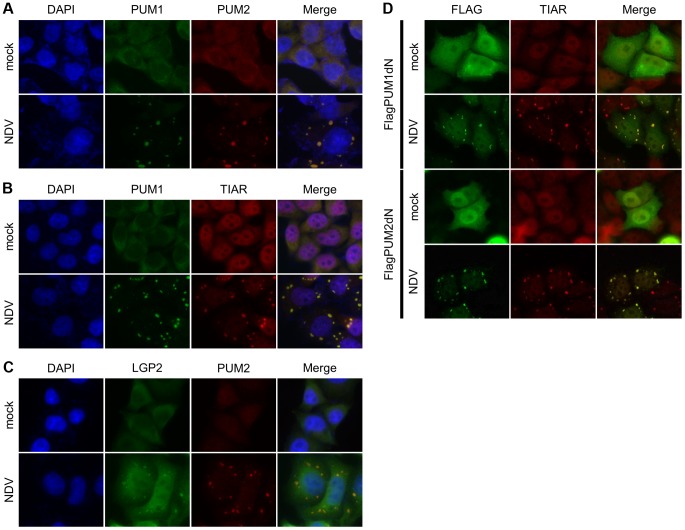
Cellular localization of PUM1, PUM2 and LGP2. (A–C) HeLa cells were mock-treated or infected with NDV for 9 h, fixed and stained with the indicated antibodies. Nuclei were stained with DAPI. (D) HEK293T cells were transfected with the expression vector for Flag-PUM1dN or Flag-PUM2dN for 48 h and mock treated or infected with NDV for 9 h. The cells were stained with anti-Flag or anti-TIAR.

It is reported that PUM1 and PUM2 are recruited to stress granules (SGs) upon stress responses, such as oxidative stress or starvation [45]. Previously, we reported that virus infection induces SG-like aggregates containing SG markers, RLR and several antiviral proteins, and termed the aggregate antiviral SGs (avSGs) [34]. avSGs are thought to function as a platform for detection of viral RNA by RLR and as action sites of antiviral proteins. We determined the cellular localization of PUM1 and PUM2 by immunostaining. In uninfected cells, PUM1 and PUM2 localized diffusely in the cytoplasm (Figure 4A). NDV infection induced co-localization of PUM1 and PUM2 into cytoplasmic speckle-like aggregates (Figure 4A). We confirmed that a SG marker, TIAR, localized with the speckles containing PUM1 in NDV-infected cells (Figure 4B); therefore, these aggregates correspond to avSG. We also confirmed that LGP2 localized in the avSG (Figure 4C)

Both N- and C-terminal domains of Pumilio proteins are required for transactivation (Figure 3E) and the N-terminal domain is responsible for interaction with LGP2. We therefore explored the function of C-terminal PUM-HD in terms of cellular localization. Flag-tagged PUM1dN and PUM2dN were expressed in cells and the cells were infected with NDV (Figure 4D). In uninfected cells, these mutants were diffusely accumulated in nuclei and cytoplasm; however, upon viral infection, these proteins localized with avSG, suggesting that PUM-HD is responsible for the localization of PUM proteins in avSG. We also determined the cellular localization of PUM1dC and PUM2dC. As shown in Figure S5D, PUM1dC diffusely localized in the cytoplasm of NDV-infected cells, whereas PUM2dC was recruited to the avSGs in response to NDV infection.

To explore the possibility that PUM1 and PUM2 are required for avSG formation, the effect of knockdown of PUM1 and PUM2 on avSG formation was examined. As shown in Figure S5A, knockdown of PUM1 or PUM2 expression did not alter avSG induction in NDV-infected cells, suggesting that PUM1 and PUM2 do not notably affect avSG assembly. We also examined the recruitment of Pumilio proteins and LGP2 in LGP2 KO cells and Pumilio knockdown cells, respectively. The knockdown of Pumilio proteins did not affect the localization of LGP2 (Figure S5B). Furthermore, Pumilio proteins were recruited to the avSGs in response to NDV in LGP2 KO cells (Figure S5C), indicating that Pumilio proteins were not involved in the recruitment of LGP2 to the avSGs and vice versa.

### PUM1 and PUM2 Augment dsRNA Binding Activity of LGP2

**Figure 5:**
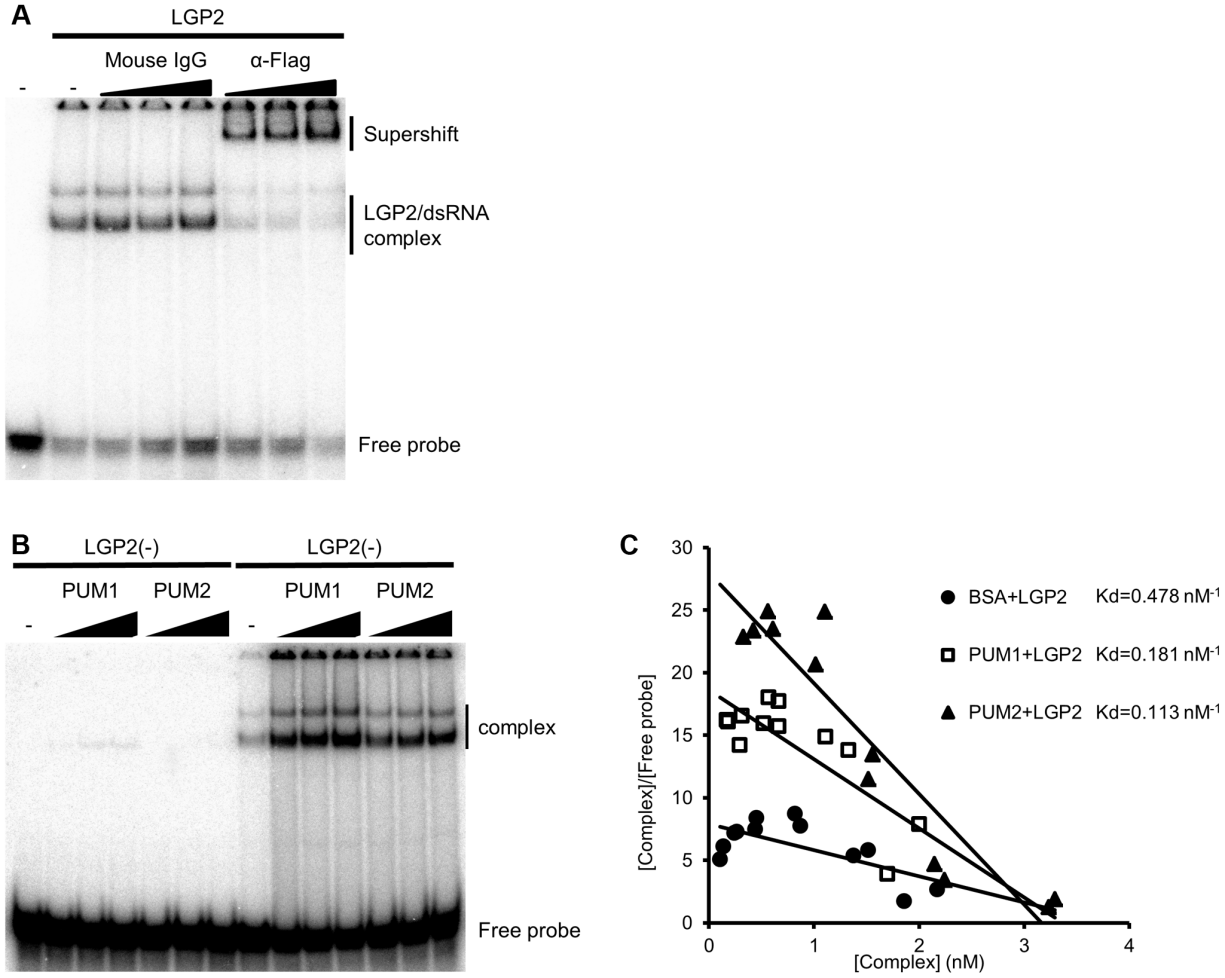
*In vitro* binding assay of dsRNA and LGP2. (A and B) Recombinant LGP2 proteins (0.125 µg) were mixed with ^32^P-labeled dsRNA in the presence of a control mouse IgG or anti-Flag antibody (0.1, 0.2 or 0.4 µg) (A) or in the presence or absence of Pumilio proteins (0.1, 0.3 or 0.5 µg) (B). The mixture were separated by acrylamide gel and the radioactivity was analyzed. (C) LGP2 dsRNA binding affinities in the absence (filled circles) or presence of PUM1 (open square) or PUM2 (filled triangle) were analyzed and the Kd values were determined.

Finally, to elucidate the mechanism of the enhancement of IFN gene expression by PUM1 and PUM2, we examined dsRNA binding activity of LGP2 in the presence or absence of PUM1 and PUM2. Electrophoresis mobility shift assay (EMSA) was performed using synthetic dsRNA and recombinant proteins. LGP2 bound to the probe, resulting in a slow-migrating complex. We confirmed that this slow-migrating band was a complex of the probe and LGP2 by a supershift experiment (Figure 5A). PUM1 and PUM2 exhibited very weak binding with the probe (Figure 5B). Interestingly, the LGP2-dsRNA complex intensity was increased with the addition of PUM1 and PUM2; however, the mobility of the complex is hardly affected. Dissociation constant for LGP2 in the absence and presence of PUM1 or PUM2 was determined by Scatchard plot analysis (Figure 5C). The Kd value indicates that PUM1 and PUM2 increased the dsRNA-binding activity of LGP2. We also purified recombinant PUM1 and PUM2 lacking PUM-HD (PUM1dC and PUM2dC) and subjected them to a binding assay (Figure S6A and B). Essentially similar results were obtained, indicating that the N-terminal domain of PUM1 and PUM2 is sufficient for increasing the dsRNA binding affinity of LGP2.

**Figure 6:**
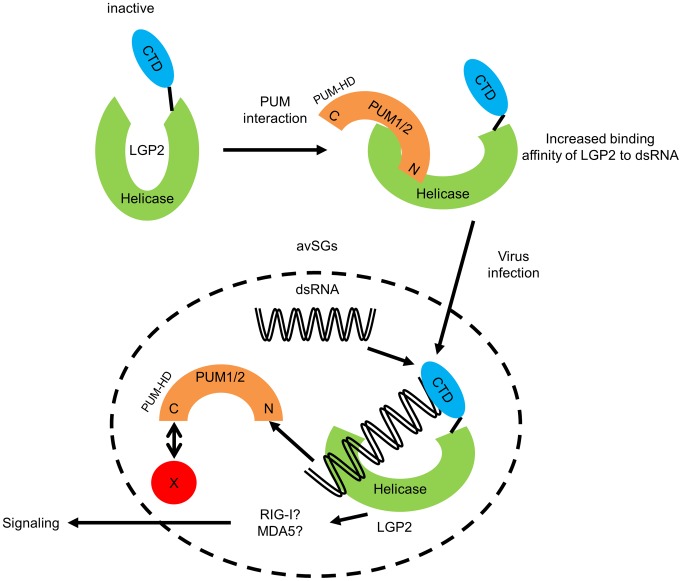
Hypothetical model for regulation of LGP2 by PUM1 and PUM2 in avSG. N-terminal domain of PUM1 and PUM2 possess intrinsic affinity to LGP2. This interaction confers higher binding affinity of LGP2 to viral dsRNA. Conformational change of LGP2 is one of the explanations for the increased affinity. Viral infection such as NDV induces avSGs and accumulation of viral dsRNA, LGP2, PUM1, PUM2 and other avSG markers into avSGs. Within avSG, dsRNA interacts with LGP2/PUM complex, producing LGP2/dsRNA complex and Pumilio proteins are released from the complex. Then, LGP2 triggers signals presumably in cooperation with RIG-I or MDA5. X: potential interacting partner of C-terminal domain of PUM1 and PUM2 determining their avSG localization.

Because the complex mobility in EMSA did not change in the presence or absence of Pumilio proteins, we examined whether the association between Pumilio proteins and LGP2 is affected in the presence or absence of dsRNA. Recombinant PUM1 and PUM2 proteins produced as GST fusion were mixed with recombinant Flag-tagged LGP2 in the presence or absence of dsRNA and pulled down with glutathione Sepharose (Figure S7). In the absence of dsRNA, we confirmed the association between PUM1 and PUM2 with LGP2; however, in the presence of dsRNA, this association was undetectable, suggesting that upon binding of LGP2 with dsRNA, PUM1 and PUM2 are released from the complex, consistent with the EMSA results. Taken together, we hypothesized that Pumilio proteins changed the conformation of LGP2 through physical associations to increase its dsRNA binding affinity (Figure 6).

## Discussion

We found that Pumilio proteins enhanced NDV-induced activation of the *IFNB* gene (Figure 1). Knockdown of PUM1 and PUM2 respectively attenuated gene activation (Figure 2), suggesting that PUM1 and PUM2 function non-redundantly. Furthermore, PUM1 and PUM2 accelerated the activation of IRFs and NF-κB transcription factors, suggesting their action in signal transduction, rather than post-transcriptional steps. Interestingly, PUM1 and PUM2 selectively interact with LGP2 but not with RIG-I, MDA5, IPS-1 or TRIM25 (Figure 3A). LGP2 knockdown diminished *IFNB* gene induction augmented by PUM1 and PUM2 (Figure 3B), suggesting that PUM1 and PUM2 augment viral RNA sensing mediated by LGP2. It was shown that LGP2 does not participate in the detection of synthetic oligonucleotides, such as poly I:C or in vitro-transcribed 5′pppRNA [23]. Consistent with this, the knockdown of PUM1 and PUM2 did not affect the IFN production induced by poly I:C, 5′pppRNA or poly dA:dT (Figure S1C). LGP2 exhibits strong binding activity to dsRNA; however, it lacks CARD, through which the signal is relayed to IPS-1. Therefore, it has been hypothesized that LGP2 cooperates with either RIG-I or MDA5. Our findings uncovered a new mechanism of sensing viral RNA by LGP2, PUM1 and PUM2.

Deletion analyses of PUM1 and PUM2 revealed that both N- and C-terminal regions are required for up-regulation (Figure 3E). The N-terminal region is sufficient for interaction with LGP2 and to increase the binding affinity to dsRNA (Figure 3D and S7). The C-terminal region, also termed PUM-HD, is sufficient for translocation to avSG upon viral infection (Figure 4D), although the underlying mechanism is unknown.

PUM1 and PUM2 have been known to regulate mRNA translation through sequence-specific recognition of NRE within the target mRNA. It was proposed that PUM-HD consists of 8 repeats of the module and each module recognizes a single nucleotide in NRE [43]. It was shown that single amino acid substitution is sufficient to abolish NRE binding and the translational regulation of PUM2 [44]. On the other hand, we found that NRE binding-deficient mutants (PUM1H972A and PUM2H850A) augment virus-induced signaling as strongly as the respective wt protein (Figure 1F), suggesting that Pumilio proteins facilitate two independent biological functions in translational and antiviral signal regulation. It is tempting to speculate that the repeated modules recognize component(s) associated with avSGs.

Concerning the molecular mechanism, we found that PUM1 and PUM2 increased dsRNA binding affinity of LGP2 (Figure 5). However, interestingly, we did not observe the ternary complex of dsRNA, LGP2 and PUM1 or PUM2; furthermore, the interaction between LGP2 and PUM1 or PUM2 was lost when dsRNA was added (Figure S7). We therefore hypothesize that PUM1 and PUM2 have a cooperative function with LGP2 through physical association to increase its binding affinity to dsRNA (Figure 6). It has been shown that LGP2 facilitates RIG-I- and MDA5-mediated signaling [23]. Also it was shown that LGP2 and RIG-I interact [46]. In light of these observations, it is probable that increased dsRNA binding of LGP2 facilitates RLR signaling.

Viral infection induces the formation of avSGs, including conventional SG markers, RIG-I, MDA5, LGP2, PKR, OAS, RNase L, DHX36, TRIM25, PUM1 and PUM2, some of which are critical in sensing non-self viral RNA and triggering antiviral signaling. Unlike SGs induced by physical stress, viral RNA is accumulated in virus-induced avSGs [34], [35], [37]. In summary, these results support the idea that avSGs act as a critical platform for sensing and discriminating viral RNA as a defense mechanism against viral infections. Although the IFN system is absent in plants, Pumilio proteins participate in the antiviral response in plants [47], suggesting that the principal mechanism of sensing non-self RNA is evolutionarily conserved.

## Materials and Methods

### Cell Culture and Reagents

L929 cells were maintained in minimal essential medium (MEM) (nacalai tesque,) containing 5% fetal bovine serum (FBS). HEK293T and HeLa cells were maintained in Dulbecco's modified Eagle's medium (DMEM) (nacalai tesque) containing 10% FBS.

### Plasmid Constructs

The p-125 Luc, p-55C1B Luc, p-55A2 Luc, pU6i and pU6i-shLGP2 have been described previously [6], [7]. pEF-Flag-PUM1 and PUM2 was obtained by subcloning cDNA into the empty vector pEF-BOS. Mutants were generated using the KOD -plus- Mutagenesis Kit (TOYOBO). TRIM25 cDNA was purchased from OriGene.

### RNAi

Negative control siRNA and siRNA targeting PUM1 or PUM2 were purchased from BONAC. siRNAs were transfected using Lipofectamine RNAiMAX (Invitrogen) according to the manufacturer's protocol. After 48 h, the cells were stimulated as indicated.

### Quantitative Real-Time PCR

Total RNA was isolated using Sepasol reagent (nacalai tesque), treated with DNase I (Roche) and subjected to reverse transcription using a High-Capacity cDNA Reverse Transcription Kit (Applied Biosystems). mRNA levels were monitored with the StepOne plus Real Time PCR System and TaqMan Fast Universal Master Mix (Applied Biosystems). TaqMan primer and probe sets for 18S rRNA, human IFNB1 and human CXCL10 were purchased from Applied Biosystems. The RNA copy numbers of the gene of interest were normalized to that of internal 18S rRNA. NDV replication levels were monitored with Fast SYBR PCR Master Mix (Applied Biosystems) using the primers specific for the NDV F gene.

### Antibodies

Anti-Flag and anti-HA antibody were purchased from Sigma and Cell Signaling Technology, respectively. Anti-GST anti-β-actin, anti-c-Myc, anti-Pumilio1, anti-Pumilio2 and anti-TIAR antibodies were from Santa Cruz Biotechnology (Santa Cruz, CA, USA). Anti-IRF-3, anti-RIG-I, anti-MDA5 and anti-LGP2 antibody were described previously [34], [48]. Alexa 488- and 594-conjugated anti-rabbit or anti-goat IgG antibodies (Invitrogen) were used as secondary antibodies.

### Immunostaining

The cells were fixed with 4% paraformaldehyde (nacalai tesque) for 10 min, permeabilized with an acetone: methanol (1∶1) solution, and blocked with 5 mg/ml bovine serum albumin (BSA) (nacalai tesque) for 30 min. The cells were incubated with the indicated primary antibodies overnight at 4°C, and then incubated with the relevant Alexa-conjugated antibodies at room temperature for 1 h. Nuclei were stained with DAPI (nacalai tesque). The cells were analyzed with a microscope (Leica microsystems).

### Luciferase Assay

Luciferase assay was performed as described previously [7]. The Dual-Luciferase Reporter Assay System (Promega) was used according to the manufacturer's protocol.

### Co-immunoprecipitation

The indicated plasmids were transfected with HEK293T cells using Lipofectamine 2000 (Invitrogen). The cell lysates were incubated with anti-Flag or anti-c-Myc antibody on ice for 30 min. The pre-washed Protein G Sepharose (GE Healthcare) was added to the mixture, which was rotated at 4°C overnight. After washing, the precipitates were eluted and separated by SPS-PAGE, followed by Western blotting.

### Viruses

NDV was grown in the allantonic cavities of 9-day-old embryonated eggs. The cells were mock treated or infected with NDV at 37°C.

### Native PAGE for IRF-3 Dimer Detection

The cell lysates were subjected to Native PAGE and Western blotting as described previously [6], [48].

### Enzyme-Linked Immunosorbent Assay (ELISA)

The cell culture supernatants were collected and subjected to ELISA with a human IFN-β ELISA kit (TORAY, Tokyo, Japan) according to the manufacturer's protocol.

### Recombinant Proteins

Recombinant LGP2 was produced as 6xHis-LGP2 fusion using baculovirus and High Five cells. 6xHis-LGP2 was bound to Ni Sepharose 6 Fast Flow (GE Healthcare), and then eluted by elution buffer containing 50 mM Tris-HCl (pH 8.0), 150 mM NaCl, 1.5 mM DTT and 500 mM imidazole.

The intact PUM1 and PUM2 were amplified by PCR and inserted into a modified pGex-6p-1 vector (GE Healthcare). The C-terminal His6-tag was inserted using a KOD plus mutagenesis kit (TOYOBO) to produce N-terminal GST and C-terminal His6 tagged proteins. The vectors were transformed into an *E. coli* BL21 (DE3) strain. Bacteria were first grown at 37°C in LB medium containing 100 µg/ml ampicillin at 160 rpm. Protein expression was induced by the addition of 0.1 mM IPTG when the absorbance at 600 nm was approximately 0.4. The cells were then grown at 16°C for 16 h at 90 rpm. The cells were harvested by centrifugation and were suspended in a lysis buffer containing 50 mM Tris-HCl (pH 8.0), 500 mM NaCl, and 20 mM imidazole supplemented with protease Inhibitor Cocktail (Roche Diagnostics) and were lysed via sonication and centrifugation. The supernatant was suspended in Ni Sepharose 6 Fast Flow (GE Healthcare), then the resin was washed with lysis buffer, and the protein was eluted by elution buffer containing 50 mM Tris-HCl (pH 8.0), 500 mM NaCl, and 500 mM imidazole. The protein was diluted by phosphate-buffered saline (PBS) and mixed with Glutathione Sepharose 4B (GE Healthcare) for 16 h. The mixture was washed with PBS and proteins were eluted by a buffer containing 50 mM Tris-HCl (pH 8.0), 150 mM NaCl, and 20 mM reduced glutathione.

### Electrophoresis Mobility Shift Assay (EMSA)

Recombinant LGP2 proteins were mixed with ^32^P-labeled synthetic dsRNA (25/25c) [49] in a reaction mixture (20 mM Tris-HCl (pH 8.0), 1.5 mM MgCl_2_, and 1.5 mM DTT) in the presence or absence of recombinant Pumilio proteins. After incubation at 37°C for 15 min, the reaction mixture was applied to a 15% acrylamide gel (TBE buffer) and the radioactivity was detected with an Image Analyzer (FUJIFILM, Tokyo, Japan).

### GST-Pull Down Assay

Recombinant LGP2 proteins were mixed with Pumilio proteins in a reaction mixture (20 mM Tris-HCl (pH 8.0), 1.5 mM MgCl_2_, and 1.5 mM DTT) in the presence or absence of synthetic dsRNA (25/25c) at 37°C for 15 min. Pre-washed Glutathione Sepharose 4B (GE Healthcare) was added to the mixture and incubated at room temperature for 1 h. After washing, the precipitates were separated by SDS-PAGE, followed by Western blotting.

## Supporting Information

Figure S1 PUM1 and PUM2 positively regulate NDV-induced IFN induction.(A-C) HEK293T cells were transfected with control siRNA or siRNA targeting PUM1 or PUM2 for 48 h. The cells were mock-treated or infected with NDV for 7, 8 or 9 h. The cell lysates were separated by Native PAGE, followed by immunoblotting with anti-pIRF-3 (A) or anti-IRF-3 (B) antibodies. The cells were infected or transfected with the indicated nucleotides for 24 h. The culture media were collected and subjected to IFN-β ELISA (C).
(PDF)

Figure S2 The knockdown of Pumilio proteins did not affect the expression level of RLRs. HEK293T cells were transfected with control siRNA or siRNA targeting human PUM1 or PUM2 for 48 h. The cells were mock-treated or treated with human IFN-β (1000 U/ml) for 24 h. The cell lysates were subjected to SDS-PAGE, followed by immunoblotting with anti-RIG-I, anti-MDA5, anti-LGP2 or anti-β-actin antibodies.
(PDF)

Figure S3 Physical interaction between PUM1 and PUM2. HEK293T cells were transfected with a HA-tagged PUM2 together with Flag-tagged PUM1. The cell lysates were subjected to IP with anti-Flag, followed by Western blotting.
(PDF)

Figure S4 PUM1 and PUM2 interacted with LGP2 through its helicase domain. HEK293T cells were transfected with a HA-tagged LGP2 full-length, helicase domain (dCTD) or CTD together with Flag-tagged PUM1 or PUM2. The cell lysates were subjected to IP with anti-Flag, followed by Western blotting.
(PDF)

Figure S5 PUM1 and PUM2 are not required for NDV-induced avSG formation. (A and B) HeLa cells were transfected with control siRNA or siRNA targeting PUM1 or PUM2. After 48 h, the cells were mock-infected or infected with NDV for 9 h. The cells were then fixed and stained with anti-TIAR and anti-NDV NP (A) or anti-TIAR and anti-LGP2 (B) antibodies. (C) LGP2 WT or KO cells were infected with NDV for 9 h. The cells were fixed and stained with anti-PUM1 and anti-PUM2 (Upper) or anti-PUM1 and anti-TIAR (Lower) antibodies. (D) HEK293T cells were transfected with Flag-tagged PUM1dC or PUM2dC for 48 h and infected with NDV for 9 h. The cells were fixed and stained with anti-Flag and anti-TIAR antibodies.
(PDF)

Figure S6 *In vitro* binding assay of dsRNA and LGP2 in the presence or absence of PUM1dC or PUM2dC. (A) Recombinant LGP2 (0.125 µg) proteins were mixed with ^32^P-labeled dsRNA in the presence or absence of Pumilio proteins lacking PUM-HD (PUM1dC and PUM2dC, 0.5 µg). The mixture was separated by acrylamide gel and the radioactivity was analyzed. (B) LGP2 dsRNA binding affinities in the absence (filled circles) or presence of PUM1dC (open square) or PUM2dC (filled triangle) were analyzed and the Kd values were determined.
(PDF)

Figure S7 Association between LGP2 with PUM1 or PUM2 in the presence or absence of dsRNA. Recombinant LGP2 proteins (0.5 µg) were mixed with Pumilio proteins (0.5 µg) in the presence or absence of dsRNA (25/25c, 0.4 µg). The mixture (10 µl) was then incubated with Glutathione Sepharose. After washing, the precipitates were eluted and separated by SDS-PAGE, followed by Western blotting.
(PDF)
