# Supplementary figures and images for: A Novel Function of Human Pumilio Proteins in Cytoplasmic Sensing of Viral Infection
Source: PLoS Pathog. 2014 Oct 23;10(10):e1004417. doi: 10.1371/journal.ppat.1004417 (PMC4207803; doi:10.1371/journal.ppat.1004417)

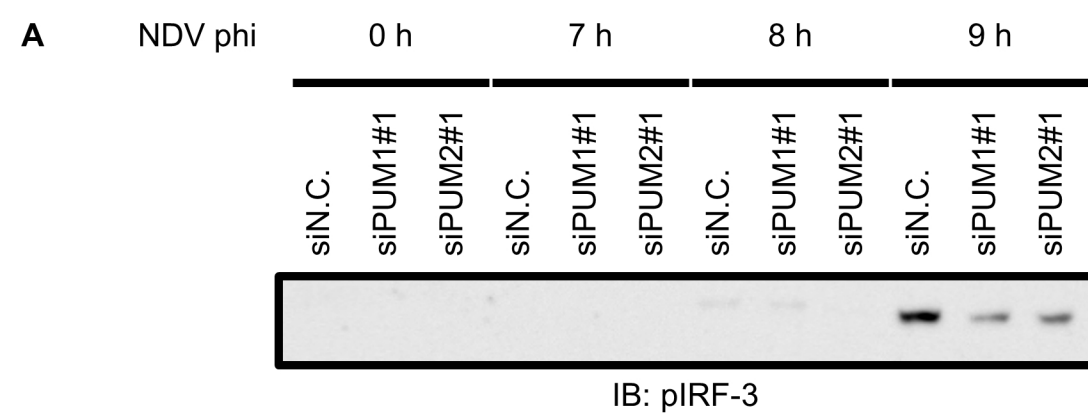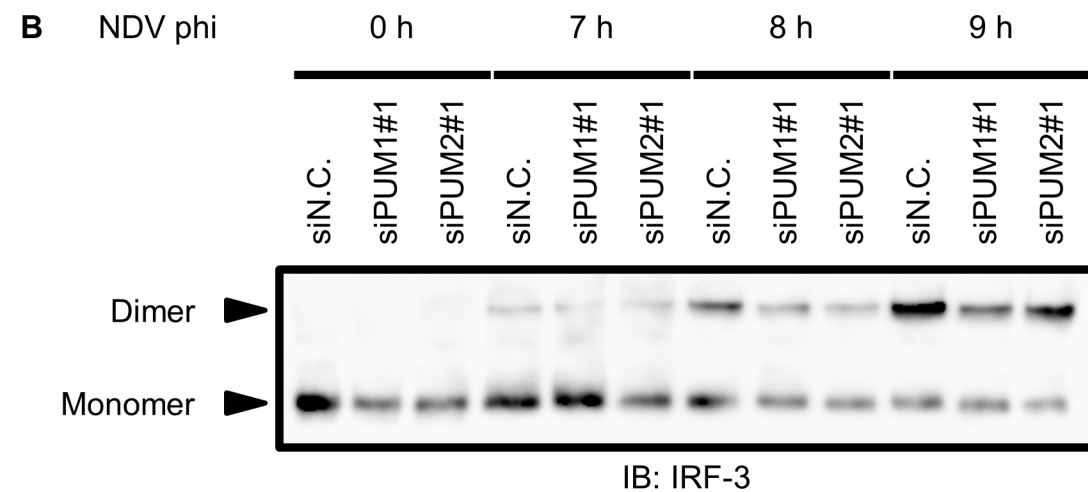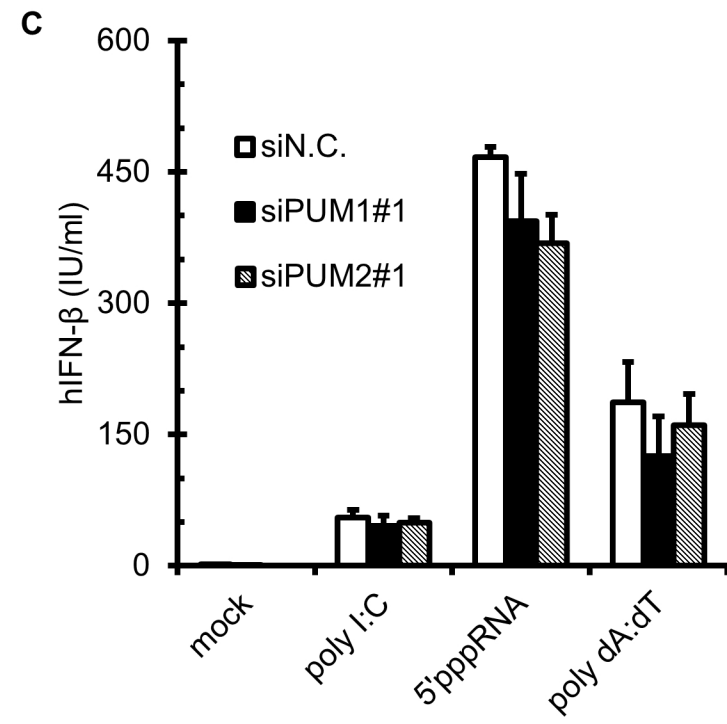

Supplement: Figure S1 — PUM1 and PUM2 positively regulate NDV-induced IFN induction.(A-C) HEK293T cells were transfected with control siRNA or siRNA targeting PUM1 or PUM2 for 48 h. The cells were mock-treated or infected with NDV for 7, 8 or 9 h. The cell lysates were separated by Native PAGE, followed by immunoblotting with anti-pIRF-3 (A) or anti-IRF-3 (B) antibodies. The cells were infected or transfected with the indicated nucleotides for 24 h. The culture media were collected and subjected to IFN-β ELISA (C). (PDF) [file ppat.1004417.s001.pdf]

IFN- $\beta$  (-)

IFN- $\beta$  (+)

siN.C.

siPUM1#1

siPUM2#1

siN.C.

siPUM1#1

siPUM2#1

IB: RIG-I

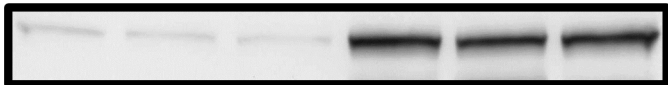

IB: MDA5

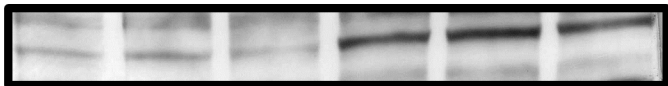

IB: LGP2

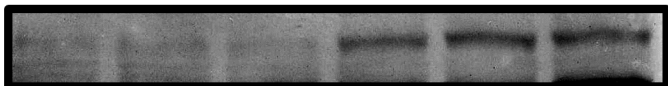

IB:  $\beta$ -actin

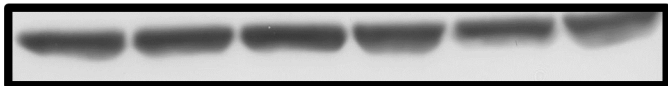

Supplement: Figure S2 — The knockdown of Pumilio proteins did not affect the expression level of RLRs. HEK293T cells were transfected with control siRNA or siRNA targeting human PUM1 or PUM2 for 48 h. The cells were mock-treated or treated with human IFN-β (1000 U/ml) for 24 h. The cell lysates were subjected to SDS-PAGE, followed by immunoblotting with anti-RIG-I, anti-MDA5, anti-LGP2 or anti-β-actin antibodies. (PDF) [file ppat.1004417.s002.pdf]

HA-PUM2

Empty

PUM1

Input (5%)

Flag

HA

IP: Flag

Flag

HA

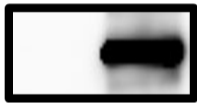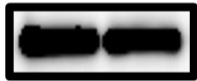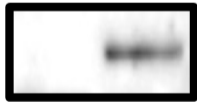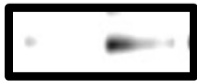

Supplement: Figure S3 — Physical interaction between PUM1 and PUM2. HEK293T cells were transfected with a HA-tagged PUM2 together with Flag-tagged PUM1. The cell lysates were subjected to IP with anti-Flag, followed by Western blotting. (PDF) [file ppat.1004417.s003.pdf]

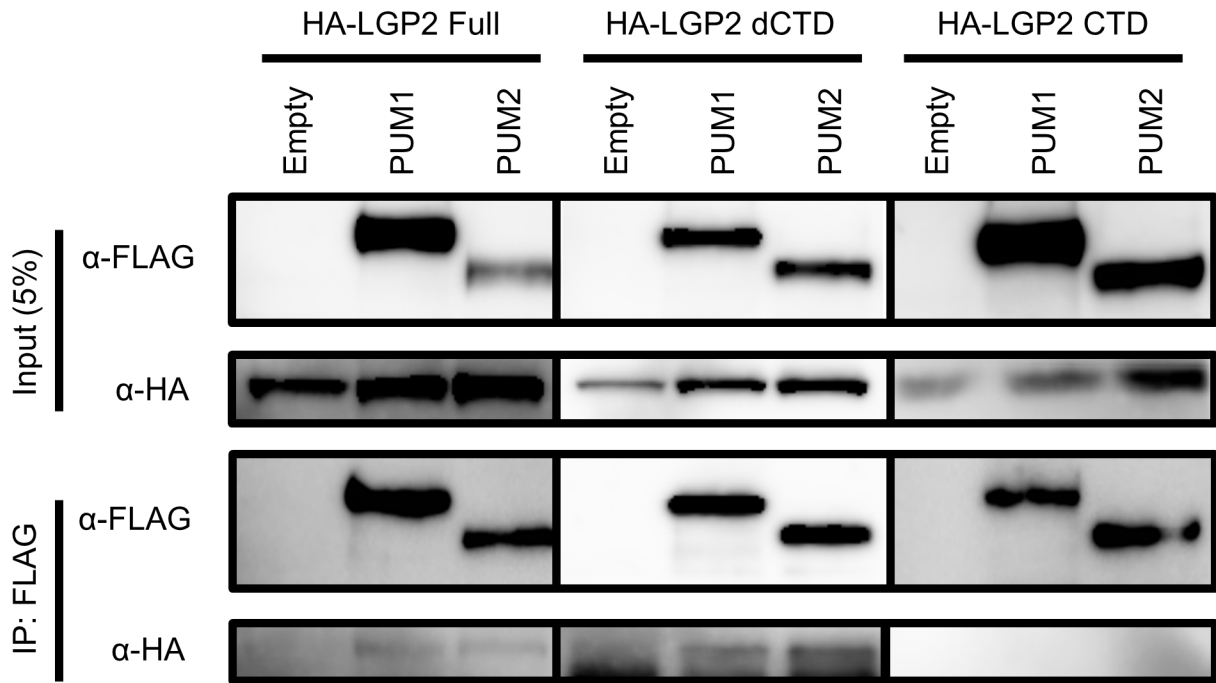

Supplement: Figure S4 — PUM1 and PUM2 interacted with LGP2 through its helicase domain. HEK293T cells were transfected with a HA-tagged LGP2 full-length, helicase domain (dCTD) or CTD together with Flag-tagged PUM1 or PUM2. The cell lysates were subjected to IP with anti-Flag, followed by Western blotting. (PDF) [file ppat.1004417.s004.pdf]

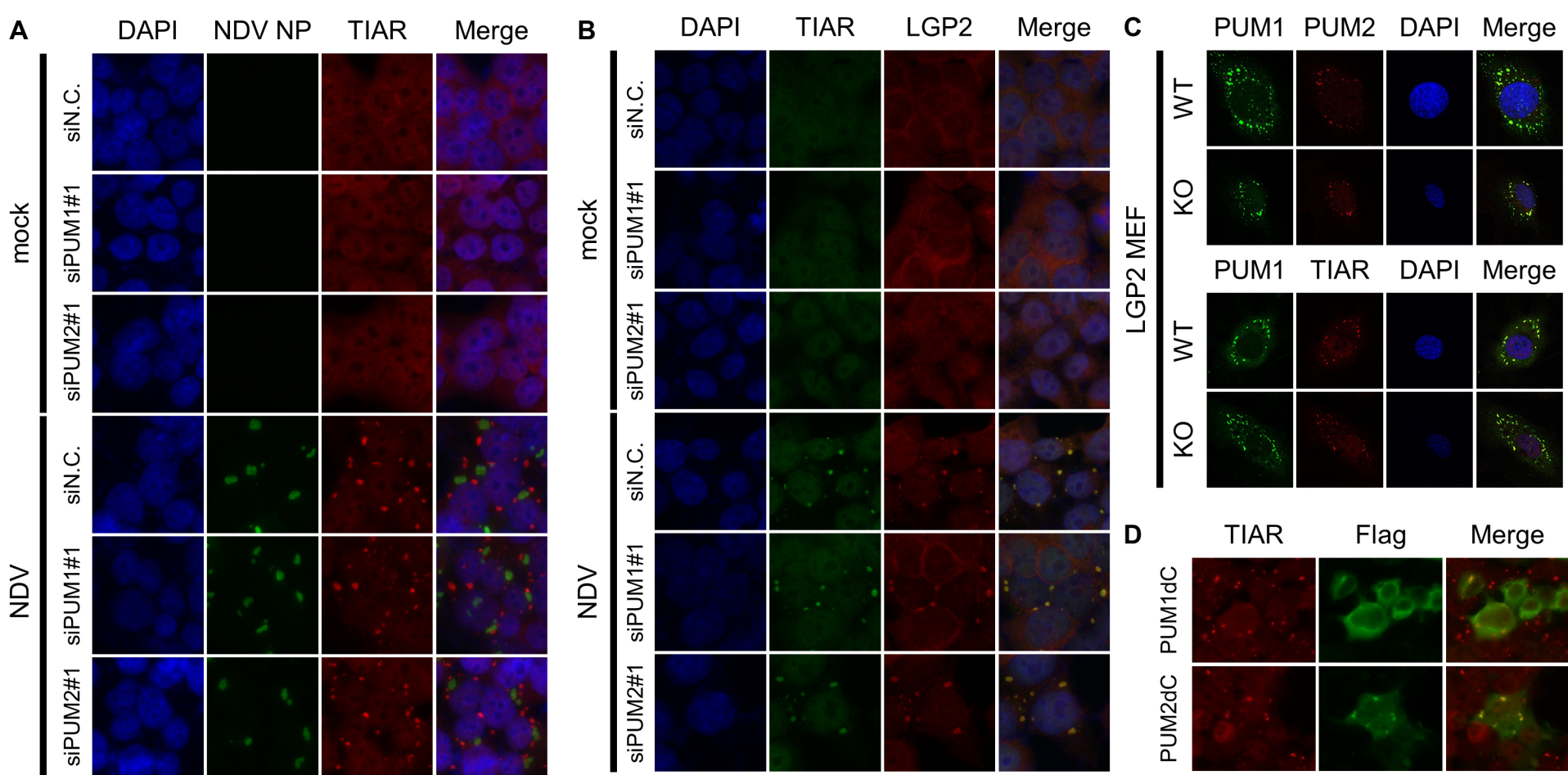

Supplement: Figure S5 — PUM1 and PUM2 are not required for NDV-induced avSG formation. (A and B) HeLa cells were transfected with control siRNA or siRNA targeting PUM1 or PUM2. After 48 h, the cells were mock-infected or infected with NDV for 9 h. The cells were then fixed and stained with anti-TIAR and anti-NDV NP (A) or anti-TIAR and anti-LGP2 (B) antibodies. (C) LGP2 WT or KO cells were infected with NDV for 9 h. The cells were fixed and stained with anti-PUM1 and anti-PUM2 (Upper) or anti-PUM1 and anti-TIAR (Lower) antibodies. (D) HEK293T cells were transfected with Flag-tagged PUM1dC or PUM2dC for 48 h and infected with NDV for 9 h. The cells were fixed and stained with anti-Flag and anti-TIAR antibodies. (PDF) [file ppat.1004417.s005.pdf]

**A**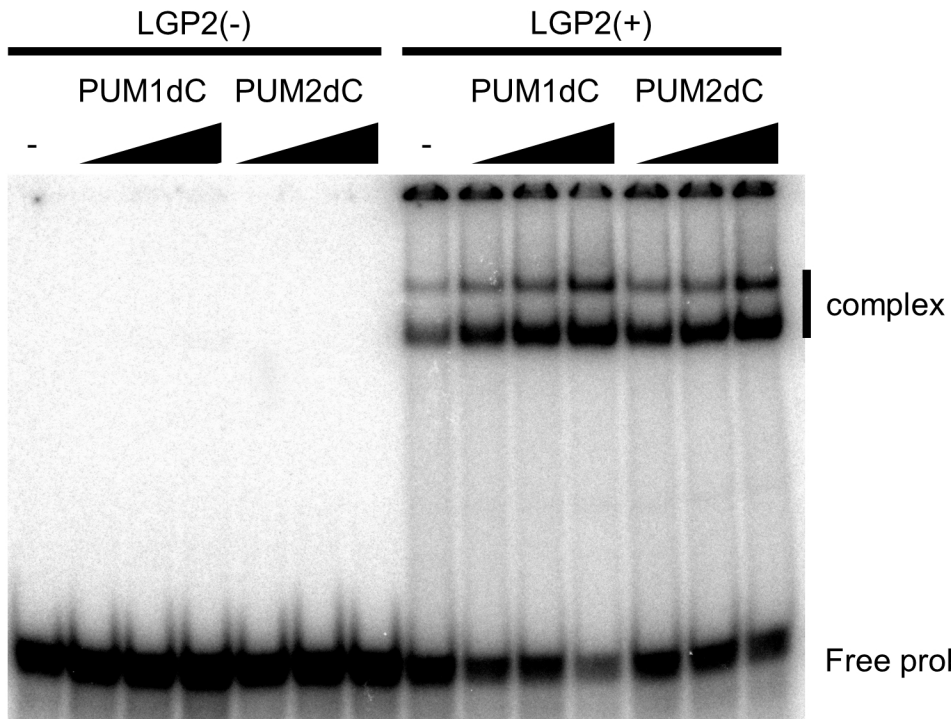**B**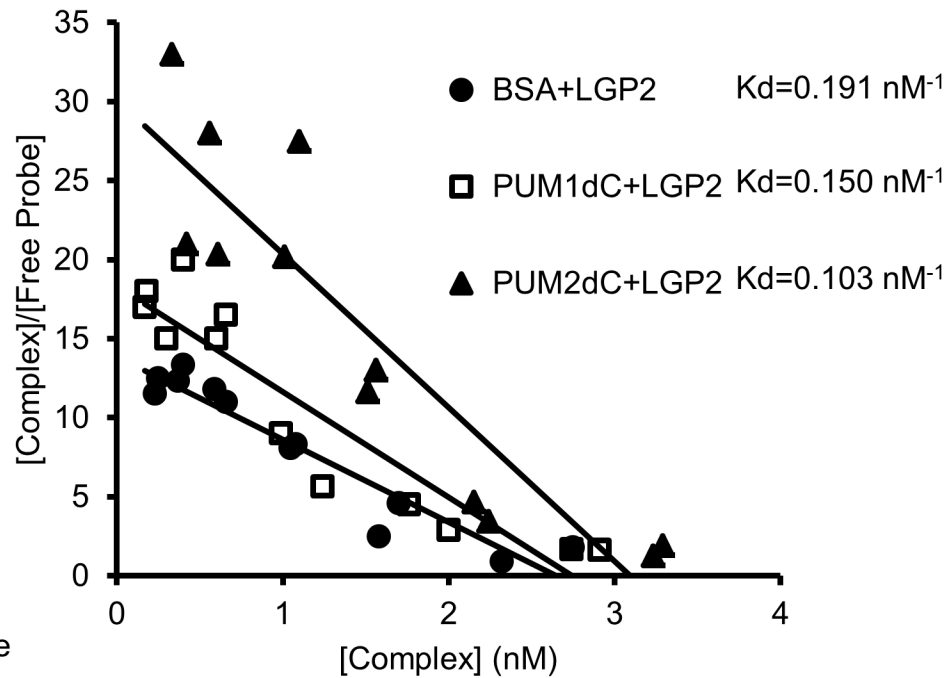

Supplement: Figure S6 — In vitro binding assay of dsRNA and LGP2 in the presence or absence of PUM1dC or PUM2dC. (A) Recombinant LGP2 (0.125 µg) proteins were mixed with 32P-labeled dsRNA in the presence or absence of Pumilio proteins lacking PUM-HD (PUM1dC and PUM2dC, 0.5 µg). The mixture was separated by acrylamide gel and the radioactivity was analyzed. (B) LGP2 dsRNA binding affinities in the absence (filled circles) or presence of PUM1dC (open square) or PUM2dC (filled triangle) were analyzed and the Kd values were determined. (PDF) [file ppat.1004417.s006.pdf]

Input

GST pull down

-

25/25c

-

25/25c

-

PUM1 PUM2

-

PUM1 PUM2

-

PUM1 PUM2

-

PUM1 PUM2

IB: GST  
(PUM1/2)

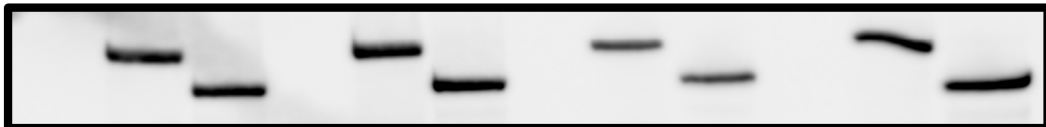

IB: Flag  
(LGP2)

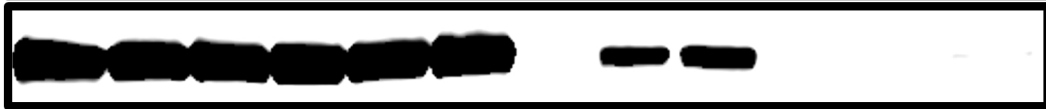

Supplement: Figure S7 — Association between LGP2 with PUM1 or PUM2 in the presence or absence of dsRNA. Recombinant LGP2 proteins (0.5 µg) were mixed with Pumilio proteins (0.5 µg) in the presence or absence of dsRNA (25/25c, 0.4 µg). The mixture (10 µl) was then incubated with Glutathione Sepharose. After washing, the precipitates were eluted and separated by SDS-PAGE, followed by Western blotting. (PDF) [file ppat.1004417.s007.pdf]
